# Hyperglycaemic Environment: Contribution to the Anaemia Associated with Diabetes Mellitus in Rats Experimentally Induced with Alloxan

**DOI:** 10.1155/2015/848921

**Published:** 2015-11-30

**Authors:** Oseni Bashiru Shola, Fakoya Olatunde Olugbenga

**Affiliations:** Department of Biomedical Sciences, Faculty of Basic Medical Sciences, College of Health Sciences, Ladoke Akintola University of Technology, Ogbomosho 210214, Oyo State, Nigeria

## Abstract

*Background*. Diabetes mellitus characterized by hyperglycaemia presents with various complications amongst which anaemia is common particularly in those with overt nephropathy or renal impairment. The present study has examined the contribution of the hyperglycaemic environment in diabetic rats to the anaemia associated with diabetes mellitus. Method. Sixty male albino rats weighing 175–250 g were selected for this study and divided equally into control and test groups. Hyperglycaemia was induced with 170 kgbwt^−1^ alloxan intraperitoneally in the test group while control group received sterile normal saline. Blood samples obtained from the control and test rats were assayed for packed cell volume (PCV), haemoglobin (Hb), red blood cell count (RBC), reticulocyte count, glucose, plasma haemoglobin, potassium, and bilirubin.* Result*. Significant reduction (*P* < 0.01) in PCV (24.40 ± 3.87 versus 40.45 ± 3.93) and haemoglobin (7.81 ± 1.45 versus 13.39 ± 0.40) with significant increase (*P* < 0.01) in reticulocyte count (12.4 ± 1.87 versus 3.69 ± 0.47), plasma haemoglobin (67.50 ± 10.85 versus 34.20 ± 3.83), and potassium (7.04 ± 0.75 versus 4.52 ± 0.63) was obtained in the test while plasma bilirubin showed nonsignificant increase (0.41 ± 0.04 versus 0.24 ± 0.06).* Conclusion*. The increased plasma haemoglobin and potassium levels indicate an intravascular haemolytic event while the nonsignificant increased bilirubin showed extravascular haemolysis. These play contributory roles in the anaemia associated with diabetes mellitus.

## 1. Introduction

Diabetes mellitus is a disorder of impaired carbohydrate metabolism resulting from a relative or an absolute deficiency of the hormone insulin. It is documented to have a global prevalence, ranking among the top causes of death in the Western world [1].

Without preference to classification, diabetes mellitus generally presents with hyperglycaemia. Hyperglycaemia is referred to as blood sugar greater than the upper reference limit for age, sex, and environmental and physiological condition [2]. In hyperglycaemic state, glucose supplies to metabolizing cells are usually impaired but not to the red blood cell. The glucose transporter on the red cell membrane,* glucose-permease*, is non-insulin-dependent; hence an excessively high concentration of red cell intracellular glucose in hyperglycaemic state is imminent [3].

Studies have shown that accumulation of intracellular glucose may increase peroxidation of red cell membrane predisposing to cell membrane defects [4]. This may influence deformability [5] as observed in the red cells of patients with diabetic retinopathy [6, 7] and also contribute to reduced blood flow in the capillaries and microcirculation as hypothesized by other research workers [8–10]. It has also been reported that effective erythropoietin synthesis may be impaired following pathologic conditions of the kidneys, contributing to the anaemia observed in diabetes mellitus [11]. These factors and several others play a role in the anaemia associated with diabetes; the effect of the hyperglycaemic environment on the red cell survival is therefore investigated by this study.

## 2. Materials and Methods

The experimental study was conducted at the Mercyland Campus of Ladoke Akintola University of Technology (LAUTECH), Osogbo. Sixty (60) white male albino rats weighing 175–250 g were acclimated for 14 days to the animal house of the Mercyland Campus of Ladoke Akintola University, Osogbo. The selected animals were housed in wire mesh, well aerated cages at normal atmospheric temperature (25 ± 5°C) and normal 12-hour light/dark cycle. They had free access to water and supplied daily with standard diet of known composition* ad libitum*. All animal procedures were in accordance with the standard recommendations for care and use of laboratory animals [12].

### 2.1. Chemicals, Reagent, and Equipment

Alloxan monohydrate was purchased from Sigma-Aldrich Chemicals Co. (St. Louis, MO, USA), protected from direct light exposure, and stored at 2–4°C. All other chemicals including stains (Leishman) were of analytical grade and obtained from licensed laboratory reagent suppliers. Machines and equipment used were properly calibrated and quality-controlled before respective analyses.

### 2.2. Induction of Diabetes

Rats were weighed and blood samples collected from the tail vein for baseline plasma glucose (glucose oxidase method) estimation using Randox glucose kit (Randox Laboratories Ltd., BT29 QY, United Kingdom). Subsequently, the animals were divided equally into two (2) groups.

Group 1 (control group) were injected with freshly prepared sterile saline. Group 2 (test group) received 170 kgbwt^−1^ alloxan preparation (2,4,5,6-tetraoxypyrimidine; 2,4,5,6-pyrimidinetetrone). All injections were done through single intraperitoneal administration using a total volume of 0.5 mL after estimating the effective dose and administered volume with respect to their weights [13].

### 2.3. Experimental Design

On days 3, 6, and 9 after injection, rats were reweighed and glucose estimation was done in all the two (2) groups described above. On day 10, twenty-four (24) rats had very high plasma glucose level greater than 250 mg/dL and were included for group 2 while four (4) lower responsive rats were excluded. Animals were sacrificed by exposure to chloroform within a closed system and blood samples were collected for the various investigations into appropriate specimen bottles. The following investigations were carried out in the course of the study: haematocrit (HCT), haemoglobin (Hb), and red blood cell count (RBC), extracted from a complete blood count analysis using SYSMEX Automated Hematology Analyzer (KX-21N, Sysmex Corporation, Chuo-ku, Kobe 651-0073, Japan); peripheral blood for reticulocyte count incubated with new methylene blue at 37°C, smeared, and estimated manually; serum total bilirubin estimated using Randox kit (Randox Laboratories Ltd., BT29 QY, United Kingdom); calorimetric method on BSA 3000 Semiautometed Biochemistry Analyser (SFRI San, Lieu dit Berganton, 33127 Saint Jean d'lllac, France); and Plasma haemoglobin according to Dacie and Lewis [14]. Plasma potassium was estimated using ISE 6000 electrolyte analyzer (SFRI San, Lieu dit Berganton, 33127 Saint Jean d'lllac, France).

### 2.4. Statistical Analysis

Data obtained were analyzed using statistical package for social sciences version 15 (SPSS Inc., Chicago, IL) for windows and expressed as mean ± 1standard deviation. Test of significance comparing control and test was done using Student's *t*-test and defined as *P* < 0.01.

## 3. Results

A total of fifty-four (54) rats, 30 controls and 24 (80%) alloxan induced hyperglycaemic tests rats, were used for this study. Two (6.6%) of the test rats were lost to death on days 2 and 3 after induction. In Table 1 we summarize the effect of alloxan administration on plasma glucose level. On day 9 after induction, significant hyperglycaemia (≥250 mg/dL) was observed in 24 (80%) rats. Four (13.3%) test rats showed no significant increase in plasma glucose after alloxan induction; there was no significant difference between the baseline glucose and glucose concentration after alloxan induction in these rats. Average plasma glucose level on day 9 after induction was significantly higher (*P* < 0.01) than the control (267 mg/dL versus 80 mg/dL). Since significant hyperglycaemia was not established in four (13.3%) of the rats, they were excluded from further studies. Data comparing the average mean values and standard deviations between parameters of the test and control group were summarized in Tables 2 and 3. In Table 2, we compare the results of plasma haemoglobin, plasma potassium, and total bilirubin concentration between the two groups. There was a significant increase (*P* < 0.01) in plasma haemoglobin and potassium concentration in hyperglycaemic rats; total bilirubin, however, was not significantly increased between the two groups although average mean was increased in the test group.  Table 3 showed the relationship between the haematocrit, haemoglobin, total red blood cell count, and reticulocyte count in the two groups. We observed a statistically significant reduction (*P* < 0.01) in haematocrit, haemoglobin, and red blood cell count among the hyperglycaemic rats; reticulocyte count was statistically higher in this group also. The red cell parameters (HCT, Hb, and RBC) were higher and stable in the control sets and reticulocyte count remained within normal limits.

## 4. Discussion

Induction of diabetes experimentally by alloxan (2,4,5,6-tetraoxypyrimidine; 2,4,5,6-pyrimidineterione) remains one of the most effective methods of establishing experimental diabetes. It is a well-known diabetogenic agent and has been widely reported to generate stable hyperglycaemia for prolong period [15]. In our study as summarized in Table 1, there was progressive induction of hyperglycaemia following alloxan administration and a stable hyperglycaemic state in 24(80%) of the alloxan induced rats. This is in consonance with several research studies that induced hyperglycaemia using alloxan [13, 16]. Misra and Aiman [17] in their study observed alloxan induced diabetes in 60% of rats using the same dosage as in our study; however they reported a dose-dependent mortality in 40% of the rats in this group. They hypothesized that susceptibility to diabetogenic and toxic effects of alloxan differs among animals of the same species. Alloxan has a narrow diabetogenic range of 160-180 kgbwt^−1^ [13]; induction therefore with a lower dose may autorevert the hyperglycaemic state following a regeneration of the pancreatic beta cells [18] while a higher dose may be cytotoxic, damaging not only the pancreatic cells but other important organs [19].

Hyperglycaemia was established on the sixth day of our study. This showed that the onset of alloxan action may be delayed [18]. Optimization of the diabetogenic agent is dependent on the dose range, route of administration, rate of injection, and age and species of experimental animal used [13, 17–20]. A study on the pharmacokinetic and pharmacodynamic profile of alloxan hypothesized unpredictable diabetes inducing alloxan effect except when administered by rapid intravenous injection [18]. Hyperglycaemic response and stability were monitored in the animals throughout the experimental process to rule out autoreversion (Table 1). Significant increase in plasma potassium and haemoglobin in the test group as depicted in Table 2 suggests episodes of intravascular red cell destruction (haemolysis within the peripheral circulation). This may be attributable to fragmentation of the red blood cells in the peripheral circulation as a result of the glucose* permease* enabled accumulated red cell intracellular glucose and generation of reactive substances, distorting the well programmed structural and functional character of the cell [3]. In addition, some red cells withstanding breakage in the circulation getting to the spleen lose deformability and are phagocytosed by the reticuloendothelial macrophages, releasing bilirubin [21]. We posit that this incidence must have informed the nonsignificant increase in total bilirubin seen in this study.

The red blood cells are clinically important haematologic cells and uniquely identified as one of the early cells affected in diabetes [22], before development of other diabetic complications. Carroll and colleagues recorded that the red cells play important role in the onset and development of several diabetic complications [23]. The mechanism underlying red cell destruction in hyperglycaemia is complex. Normal erythrocytes are biconcave shaped cells, measuring about 8 *µ*m in diameter, with an average volume of 90fL and surface area of 140 *µ*m^2^. According to Mohandas and Gallagher, red blood cell has a membrane which is highly elastic, rapidly responds to applied fluid stress, and is stronger than steel in terms of structural resistance [24]. Despite this unique feature, a slight alteration in structural composition, small increased surface area, haemoglobin hyperviscosity, and autooxidation, amongst others, poses a challenge to the oxygen transporting cell and results in cell lysis [25, 26]. In the process of performing oxygen transport function, RBCs are exposed to high level of endogenous and exogenous oxidative metabolites [27]. These accumulating reactive substances potentiate complex oxidative processes with severe damaging consequence on the cell membrane, structure, and function. However, to optimize their exclusive role as well as to survive the rigors of circulation, the highly specialized blood cells have evolved an extensive array of enzymatic and nonenzymatic antioxidants systems, including membrane oxidoreductases, cellular antioxidants such as catalase and superoxide dismutase (SOD), and enzymes that continuously produce reducing agents through the glutathione (GSH) system [28]. In hyperglycaemic state, generation of reactive oxidative substances is markedly increased creating a redox imbalance within the red cell environment and limiting the cell antioxidative potential [22, 29]. Tiwari and Ndisang reported that glucose mediated increase in reactive oxygen species is one of the biochemical changes associated with type 1 diabetes (enhanced hyperglycaemia-mediated oxidative stress) [30]; other biochemical reactions associated with hyperglycaemia are diacylglycerol production and subsequent activation of the protein kinase C pathway, flux through the polyol metabolic pathway, secretion of cytokines, and modification of proteins and lipids that becomes nonenzymatically glycated forming Schiff bases and amadori products with resultant, irreversible generation and accumulation of glycated end products [31–35]. The red cells demonstrating an unregulated access to glucose uptake are in this state exposed to high glucose concentration both intracellularly and within the vascular environment [35]; this increases glucose oxidation and accumulates glucose metabolites, including NADPH which promotes susceptibility to lipid peroxidation, membrane damage, and intravascular cell death [4]. One of the greatest challenges to the well-equipped red cell antioxidant system as documented by Mohanty and colleagues is the increased autoxidation of haemoglobin (Hb) bound to the membrane in hyperglycaemic state which is relatively inaccessible to the antioxidant system [36]. Besides haemoglobin, ROS also critically affects other proteins in the red cell since they are easy target of ROS, majorly the spectrin, ankyrin, actin, and protein 4.1 [37]. Oxidation of biomolecules at amino acid active sites can also trigger rapid deactivation of enzyme and shut down the antioxidant system [22]. RBCs thus become highly susceptible to oxidative damage from accumulated reactive substance generation. A number of* in vitro/in vivo* studies have shown that several RBCs parameters are negatively affected by increased oxidative stress as observed in diabetes [38–41]. One of these is an assessment of heme degradation products (HDP) to determine the red cell oxidative status which was increased in older RBC as they tend to senescence [36]; this was also observed in RBC of diabetics [42] suggesting reduced membrane deformability [23]. In addition, oxidative stress also inhibits Ca-ATPase, responsible for regulating the intracellular concentration of calcium [43, 44]. With increased intracellular calcium, the Gardos channel is activated causing leakage of intracellular potassium; this alters cation homeostasis resulting in cell shrinking and lysis [45] as described in our study. Besides the red cell are the vascular endothelial cells with high amount of glucose transporter and also an unrestricted access to glucose in-flow [35]. Vascular complications in diabetes are associated with formation of cross-links between key molecules in the basement membrane of ECM and eruption of basement membrane lesions; this results in thickening of the blood vessels subjecting the already weakened red blood cells to fragmentation and contributing to premature destruction of the red cell in circulation [46]. Hence, red cell fragmentation and intravascular haemolysis are common events associated with damaged blood vessels, especially within the microvascular environment (microangiopathy).

Despite the undoubted fact that hyperglycaemia battles all the tissues in the body, it is established that diabetic complications are observed in a subset of cell types; capillary endothelial cells in the retina, mesangial cells in the renal glomerulus, and neurons and Schwann cells in peripheral nerves. Brownlee explained that, in hyperglycaemic state, most cells reduce transport of glucose inside the cell so that their internal glucose concentration stays constant. However, the cells damaged by hyperglycemia, including the red blood cells, are those that cannot do this efficiently because glucose transport rate does not decline rapidly, leading to high glucose inside the cell [35]. In view of this we propose that oxidative stress distorted biochemical processes and impaired deformability and cell membrane weakness, fragmentation, and intravascular and extravascular destructions; as observed in our study are the features characterizing the onset of anaemia associated with type 1 diabetes.

Anaemia stimulated hypoxia has been shown to increase hypoxia-inducible factor 1 which promotes synthesis of erythropoietin, inducing reticulocytosis [47]. The bone marrow responsiveness to the haemolysis through significant reticulocytosis indicates that alloxan toxicity has no destructive effect on the bone marrow at dosage used. It is further established that anaemia observed in earlier diabetes is contributed to by intravascular and extravascular haemolysis while anaemia of chronic long standing diabetics is caused by renal pathology [48].

In conclusion, we infer from our study that red cell destruction due to hyperglycaemic environment is predominantly intravascular with minor contribution from the extravascular environment and the presenting anaemia is a responsive type differing from the nonresponsive chronic anaemia associated with diabetic nephropathy documented by other workers.

## Figures and Tables

**Table 1:** The effect of alloxan on plasma glucose level.

Control (*n* = 30)		Test (*n* = 28)	
Saline injection		Alloxan injection	
Days	Average plasma glucose (mg/dL)	Days	Average plasma glucose (mg/dL)
0	79	0	79
3	82	3	102
6	80	6	207
9	80	9	267
12	79	12	308

**Table 2:** Table of significance comparing test and control plasma haemoglobin, potassium, and bilirubin concentration.

Subject	Plasma Hb (mg/L)	Plasma K^+^ (mmol/L)	Plasma total bilirubin (mg/dL)
Test mean (*n* = 24)	67.50 ± 10.85	7.04 ± 0.75	0.41 ± 0.04
Control mean (*n* = 30)	34.20 ± 3.83	4.52 ± 0.63	0.24 ± 0.06
*t*-test	26.4	11.52	0.28
*P* value	<0.01	<0.01	>0.01

**Table 3:** The test of significance of hyperglycaemia on PCV, Hb, RBC, and reticulocyte count.

Subject	PCV (%)	Hb (g/dL)	RBC (×10^12^/L)	Retics. count (%)
Test mean (*n* = 24)	24.40 ± 3.87	7.81 ± 1.45	3.47 ± 0.29	12.4 ± 1.87
Control mean (*n* = 30)	40.45 ± 3.93	13.39 ± 0.40	7.16 ± 0.25	3.69 ± 0.47
*t*-test	−13.04	−16.61	−42.77	−20.19
*P* value	<0.01	<0.01	<0.01	<0.01
